# Microbial Oil Production by *Yarrowia lipolytica* Under Semi-Continuous Cultivation and Potential Utility of Spent Supernatant

**DOI:** 10.3390/foods15071245

**Published:** 2026-04-05

**Authors:** Şuheda Uğur, Bartłomiej Zieniuk, Magdalena Górnicka, Dorota Nowak, Agata Fabiszewska

**Affiliations:** 1 Department of Chemistry, Institute of Food Sciences, Warsaw University of Life Sciences-SGGW, 159c Nowoursynowska St., 02-776 Warsaw, Poland; agata_fabiszewska@sggw.edu.pl; 2 Department of Human Nutrition, Institute of Human Nutrition Sciences, Warsaw University of Life Sciences-SGGW, 159c Nowoursynowska St., 02-776 Warsaw, Poland; magdalena_gornicka@sggw.edu.pl; 3 Department of Food Engineering, Institute of Food Sciences, Warsaw University of Life Sciences-SGGW, 159c Nowoursynowska St., 02-776 Warsaw, Poland; dorota_nowak@sggw.edu.pl

**Keywords:** energy recovery, semi-continuous culture, renewable bioresources, single-cell oil, single-cell protein, *Yarrowia lipolytica*

## Abstract

Microbial oil production has gained attention as a sustainable and cost-effective alternative to conventional vegetable and fish oils. Among oleaginous microorganisms, *Yarrowia lipolytica* is notable for its ability to accumulate lipids exceeding 20% of its dry weight. This study aimed to evaluate semi-continuous cultivation as a strategy for sustainable microbial oil production by *Y. lipolytica*, while also assessing the potential utility of the spent supernatant. Three different feeding frequencies were evaluated. In the 24 h feeding regime, the maximum oil concentration reached 11.22 g/L, decreasing to 8.43 g/L by the 88th hour. In the 6–6–12 h feeding strategy, crude protein content peaked at 43.75% of dry mass at 22 h. Fatty acid profiling revealed consistently low saturated fatty acid (SFA) levels (4.93–10.30%), while unsaturated fatty acids (UFA) dominated (89.69–95.05%). Monounsaturated fatty acids (MUFA) were predominant, reaching up to 81.24%, whereas polyunsaturated fatty acids (PUFA) ranged from 20.78% to 29.98%. Oleic acid was the most abundant fatty acid across all conditions. This composition supports the potential of microbial oil from *Y. lipolytica* as a sustainable alternative edible lipid ingredient for human food applications, complementing conventional plant-based oils. The favorable unsaturated fatty acid profile indicates its potential suitability for incorporation into food formulations requiring nutritionally desirable lipid sources. As part of the sustainability-oriented approach of the study, the freeze-dried post-culture supernatant was also evaluated for its potential further utilization. With a calorific value of 10.43 kJ/g and significant phosphorus and potassium levels, it shows potential as a biofuel feedstock and as a biofertilizer or biostimulant.

## 1. Introduction

Microorganisms are single-celled organisms that include many types, such as bacteria, fungi, yeast, and algae. These organisms contain lipids that form the basis of their cell membranes, typically constituting 6–8% of their dry weight. These lipids play roles in energy storage and cell signaling. Microorganisms that produce more than 20% of their dry weight as lipids are called “oleaginous”. The oils produced by these microorganisms are now commonly referred to as “single-cell oils” (SCOs) [1]. Microbial oils produced by *Yarrowia lipolytica* have attracted considerable interest due to their potential applications in the food, feed, biofuel, and oleochemical industries. Their production also represents a sustainable alternative to conventional lipid sources, particularly when low-cost or waste-derived substrates are used [1]. The term “SCO” was coined by Ratledge in 1974 to describe the lipids of microorganisms and was initially used to suggest that these lipids could be equivalent to commercial vegetable and animal oils [2]. Over time, the term has broadened to include more complex lipids, such as algal lipids, even in cases where triacylglycerols are not predominant [2]. In today’s health-conscious society, where nutritional awareness is increasingly prioritized, the significance of essential fatty acids such as omega-3, i.e., docosahexaenoic acid (DHA) and eicosapentaenoic acid (EPA), omega-6, and omega-9 has gained importance. Since these fatty acids cannot be synthesized by the human body, they must be obtained from food [3]. Foods such as oily fish (e.g., salmon), walnuts, flaxseeds, and chia seeds contain high amounts of omega-3 fatty acids [4,5]. Common sources of omega-6 are plant-based oils like sunflower oil and soybean oil [6]. Furthermore, omega-9 monounsaturated fatty acids (MUFAs), like oleic acid, reduce low-density lipoprotein (LDL) cholesterol, improve insulin balance, and support cell membrane integrity [7,8]. Depending on the importance of these fatty acids, many alternatives, other than vegetable and fish oils, have been developed. In parallel, increasing sustainability concerns and supply pressures on conventional edible oils have accelerated interest in scalable lipid sources that can be integrated into human food systems. In this context, single-cell oils are increasingly considered not only as biotechnological products, but also as potential edible lipid ingredients for food formulations, provided that their composition and quality meet nutritional and technological requirements.

One such alternative is microbial oils produced by single-celled organisms like bacteria, yeasts, fungi, and microalgae [9,10,11]. Among oleaginous microorganisms, *Y. lipolytica* stands out for its high lipid accumulation capacity and ability to metabolize lipid derivatives, attracting attention in natural and industrial settings. This yeast species presents significant potential for biotechnological research aimed at identifying alternative oil sources [12]. Microbial oil production (SCO), although an alternative to plant-based and fish oils, is not yet widely used due to the high production cost [13]. A comparative overview of the fatty acid composition of selected oleaginous microorganisms is presented in Table 1 [14,15,16,17,18,19].

Plant-based oils are advantageous because they can be produced with a lower budget [11]. However, plant oil production requires large amounts of water and arable land, which increases production costs. Some strategies, such as the use of waste substrates, have been developed in microbial oil production to reduce production costs and support sustainability. For example, waste materials obtained from the food and agricultural industries can serve as a carbon source for microbial growth, thereby reducing the cost of the carbon source required for the growth of microorganisms [20]. Moreover, the cultivation of oleaginous yeasts can be conducted in bioreactors, making it relatively easy to scale, and it does not require light for biomass growth, unlike microalgae. These microorganisms have minimal nutritional requirements, and their cultivation is unaffected by climate or seasonal variations, making them a sustainable alternative to traditional plant oils [9]. Fed-batch culture mode is widely employed in biotechnology and microbial fermentation processes. Instead of adding all the nutrients to the medium at the start, nutrients are introduced in intervals (fed-batch mode) or continuously throughout the culture (continuous mode) [21]. This method optimizes cell growth and product yield while avoiding the inhibitory effects of high nutrient concentrations [22]. Semi-continuous culture is more effective in achieving high cell density and enhancing microbial oil production. Periodical nutrient addition allows cells to grow without nutrient limitation and ensures the efficient use of the carbon source, thereby significantly increasing microbial oil production [23]. In this context, the *Y. lipolytica* KKP 379 strain is noteworthy for its ability to produce lipids using waste oils and other industrial by-products [24]. During the cultivation process aimed at producing biomass with a high lipid content, a significant amount of supernatant is obtained after the biomass is separated from the medium. The drying and reuse of the supernatant formed after the microorganisms have utilized the carbon source both reduce waste and promote sustainability. These approaches help lower microbial oil production costs, making them a more sustainable alternative [25]. The supernatant obtained in microbial oil production can be used alternatively in many ways [26]. The waste supernatant needs drying to reduce moisture and is mixed with binding agents to form a homogeneous blend [25,26]. The mixture is shaped under high pressure using pelletizing or briquetting machines, cooled, and quality control. Coatings or additives can be applied if necessary to enhance water resistance or improve combustion properties [27,28]. Converting dried microbial supernatants into bio-briquettes or bio-pellets provides a carbon-neutral energy source that reduces dependence on fossil fuels and minimizes environmental impacts [12].

This study aimed to produce cost-effective, sustainable microbial oil by cultivating *Y. lipolytica* on waste media. Post-frying oil, a low-cost carbon source, was utilized in semi-continuous cultivation to optimize lipid synthesis and reduce reliance on conventional media. Instead of batch cultures, the semi-continuous mode of action was preferred, which allowed periodical nutrient supply to the yeast culture, resulting in more significant lipid accumulation and biomass. Additionally, to maximize yeast biomass and lipid synthesis, three different strategies for introducing additional portions of the medium were explored, and the fatty acid profiles of cellular lipids and residual oil were analyzed to examine the efficiency and quality of lipid accumulation. Research focused on optimizing microbial oil production is not only a matter of biotechnological interest but also directly relevant to food science, as it supports the development of sustainable, nutritionally suitable alternative edible oils for human consumption. Given its nutritionally favorable fatty acid profile, the microbial oil produced in this study may be considered as a potential edible lipid ingredient for human foods, with prospective use in food formulations as an alternative or complementary oil source.

## 2. Materials and Methods

### 2.1. Yeast Strain

The *Y. lipolytica* KKP 379 wild-type strain was obtained from the Collection of Industrial Microorganisms at the Prof. Wacław Dąbrowski Institute of Agricultural and Food Biotechnology State Research Institute in Warsaw, Poland. The strain was preserved at −20 °C in a 20% (*v*/*v*) glycerol solution within YPG nutrient broth containing (in 1.0 L: yeast extract, 10.0 g; peptone, 20.0 g; glucose, 20.0 g; pH 5.0) [29].

### 2.2. Culture Conditions

**Figure 1:**
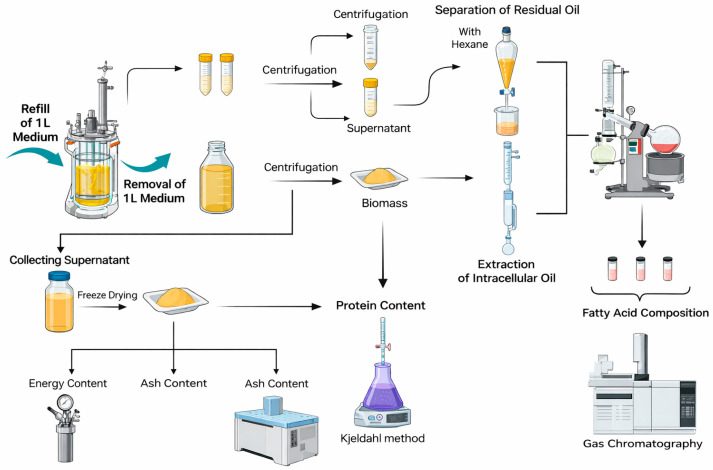
Schematic representation of semi-continuous cultivation and supernatant utilization in *Y. lipolytica*. Created in https://BioRender.com.

Inoculum shaken culture was performed in 100 mL of YPG medium and incubated for 20 h at 28 °C in an IKA KS 4000 ic control shaker (IKA company, Konigswinter, Germany) at 150 rpm. Semi-continuous cultures were carried out in a BIO FLO 3000 bioreactor (New Brunswick, Hamburg, Germany) at 28 °C and 0.025% (*v*/*v*) inoculum. The initial (and maintained) working volume was 4.0 L. The medium was aerated with compressed air at a flow of 105 L/h/L medium (1.75 vvm). Mineral media with 50 g/L post-frying waste oil were prepared for bioreactor cultures. The waste frying oil (WFO) used in this study was collected from a local university canteen and was identified as post-frying rapeseed oil. Mineral media contained g/L: yeast extract, 2.0; peptone, 1.0; (NH_4_)_2_SO_4_, 2.5; KH_2_PO_4_, 7.0; Na_2_HPO_4_, 2.5; FeSO_4_ × H_2_O, 0.16; CaCl_2_, 0.15; MnCl_2_ × 4H_2_O, 0.08; ZnSO_4_, 0.02. A pH glass electrode was used to determine the pH of the culture. During bioreactor cultivation, the agitator speed was adjusted from 200 to 600 rpm under cascade control to maintain dissolved oxygen (DO) ≥ 30% (air saturation). Three different experiments were performed in 3 different medium exchange frequencies during the day: 12–12 h, 6–6–12 h, and 24 h intervals (after an initial batch phase of 0–16 h). For each feeding frequency, 1.0 L of culture broth was withdrawn and replaced with 1.0 L of fresh medium, corresponding to a 25% exchange fraction per event (Figure 1). Kinetic parameters of *Y. lipolytica* bioreactor cultures were calculated according to Fabiszewska et al. [29] at the selected sampling times as follows.

Biomass concentration (Equation (1)):

(1) $$X(g/L)=\frac{m_{dry\;biomass}}{V}$$

where m_dry biomass_ is the dry weight of biomass (g), and V is the culture volume (L).

Storage lipid yield per biomass (Equation (2)):

(2) $$Y_{L/X}(g/g)=\frac{m_{lipids}}{m_{dry\;biomass}}$$

expressed as the mass of storage lipids per mass of dry biomass.

Concentration of cellular lipids (Equation (3)):

(3) $$L_{max}(g/L)=X\times Y_{L/X}$$

where L_max_ is the maximum concentration of cellular lipids in the culture broth (g/L).

### 2.3. Determination of Residual Oil

From the bioreactor, 90 mL samples were collected at specific intervals using the semi-continuous culture method and placed into two separate Falcon tubes. The collected samples underwent centrifugation (10,000 rpm for 10 min, 4 °C), repeated three times to ensure complete separation. Following each centrifugation cycle, the oil phase within the Falcon tubes was carefully collected and transferred to another container. Subsequently, 10 mL of hexane was added to the collected sample to facilitate oil dissolution. The mixture was thoroughly homogenized and then subjected to a separation process. The hexane-enriched oil phase was isolated once the solution formed two distinct phases. This extraction procedure was performed three consecutive times to ensure maximal oil recovery. Magnesium sulfate (MgSO_4_) was then added to the hexane-oil solution to dry the extract. The solution filtered on filter paper was ready for evaporation. Hexane was evaporated using the Büchi Rotavapor R-200 (Büchi AG, Flawil, Switzerland). Then, the residual oil ratio remaining in the flask was calculated. The residual oil measured by this method was considered to represent the unconsumed oil remaining in the fermentation broth.

### 2.4. Freeze Drying of Biomass and Supernatant

The yeast biomass was separated from the supernatant using a high-speed centrifuge (8000 rpm, 20 °C, 8 min; Sigma, Osterode am Harz, Germany) and washed twice with distilled water in order to remove residual waste oil. The collected biomass was divided and transferred into Petri dishes with a diameter of 90 mm. The dishes were then frozen at −40 °C using an Irinox freezer (Corbanese, Italy). Following this, the samples were freeze-dried in a Christ Gamma 1–16 apparatus (Osterode am Harz, Germany). The material was placed on shelves at 0 °C, and secondary drying was performed at 10 °C. The same procedure was applied for freeze-drying of supernatant, which was firstly 10× concentrated with Büchi Rotavapor R-200 (Büchi AG, Flawil, Switzerland) at 60 mbar.

### 2.5. Determination of Intracellular Lipids

Freeze-dried biomass samples were placed in filter paper and subjected to Soxhlet extraction using *n*-hexane as the solvent. A dry biomass-to-solvent ratio of 1:15 (*w*/*v*) was used. The extraction was carried out for 2 h, corresponding to approximately 12 Soxhlet cycles. After extraction, the solvent was evaporated, and the lipids were measured gravimetrically. The intracellular lipid content was calculated based on the mass of lipids recovered after solvent evaporation in relation to the dry biomass used for extraction.

### 2.6. Analysis of Fatty Acid Composition

Gas chromatography was used to determine fatty acid components. In preparing fatty acid methyl esters (FAME), the sodium methoxide transesterification method was used following PN-EN ISO 5509:2001 [30,31]. The YL6100 GC chromatograph was equipped with a flame ionization detector and a BPX-70 capillary column with a length of 60 m, a film thickness of 0.25 µm, and an inner diameter of 0.25 mm. The oven was held at 70 °C for 30 s; then the temperature was increased by 15 °C per minute to 160 °C; it was increased by 1.1 °C per minute from 160 °C to 200 °C and stabilized at 200 °C for 12 min; then it was increased by 30 °C per minute from 200 °C to 225 °C and stabilized at 225 °C for 60 s. The injector temperature was set at 225 °C, the split ratio was 1:50, and the detector temperature was set at 250 °C. Nitrogen flow at a 1 mL rate was used as the carrier gas. The obtained results determined each FA’s percentages (% FA peak area was calculated). FAs were compared with the retention times of FAME peaks with the FAME standard (Supelco 37 Component FAME Mix, Sigma-Aldrich Inc. (Supelco), St. Louis, MO, USA) [30].

### 2.7. Crude Protein Content

Crude protein content in the freeze-dried biomass and freeze-dried supernatant was determined by the Kjeldahl method based on total nitrogen content. A 0.5 g sample was subjected to the mineralization with 5 mL of H_2_O_2_ and 15 mL of H_2_SO_4_ for a 1.5 h incubation to achieve complete digestion. After mineralization, the resulting solution was transferred into a volumetric flask, and deionized water was added up to the calibration mark to dilute the sample. For ammonia distillation, 10 mL of the mineralized sample solution was transferred to a Parnas–Wagner distillation apparatus and mixed with 10 mL of NaOH. Three drops of phenolphthalein were added, and a small amount of water was used to ensure sufficient volume for distillation. The released ammonia was distilled into 10 mL of boric acid (H_3_BO_3_) solution containing eight drops of a red indicator. Distillation continued until the solution leaving the condenser became neutral (pH 7). The distillate was then titrated with 0.1 M HCl until the color changed from green to pink. The nitrogen content was calculated from the volume of HCl used for titration, and the crude protein content was determined using a nitrogen-to-protein conversion factor of 6.25.

### 2.8. Ash Content

The ash content in the freeze-dried supernatant was measured by combusting the material in a muffle furnace. This process involved taking a specific quantity of the sample, placing it in a pre-dried and weighed porcelain crucible, and combusting it in an air atmosphere at temperatures exceeding 500 °C. After the combustion, the crucible was allowed to cool to room temperature in a desiccator before being weighed again.

### 2.9. Energy Content

The heat of combustion was measured using the bomb calorimetry method in accordance with ISO standards 1928:2009 [32]. The analysis was conducted with a KL-12Mn bomb calorimeter (PRECYZJA-BIT, Bydgoszcz, Poland).

### 2.10. Reducing Sugar Content

Reducing sugars in the freeze-dried supernatant were determined using the 3,5-dinitrosalicylic acid (DNS) method. The concentration was calculated from a glucose calibration curve, and the results were expressed as mg glucose equivalents per g dry weight (DW).

### 2.11. Elemental Compositions of Freeze-Dried Supernatant

The total amounts of carbon, nitrogen, and sulfur in the freeze-dried supernatant were measured through dry combustion utilizing the Vario MacroCube elemental analyzer (Elementar, Langenselbold, Germany). The total concentrations of phosphorus (P), potassium (K), sodium (Na), magnesium (Mg), calcium (Ca), manganese (Mn), iron (Fe), zinc (Zn), aluminum (Al), chromium (Cr), nickel (Ni), arsenic (As), boron (B), lead (Pb), titanium (Ti), copper (Cu), strontium (Sr), zirconium (Zr), barium (Ba), vanadium (V), and cobalt (Co) were determined using inductively coupled plasma atomic emission spectrometry (ICP-OES) with an Avio 200 system from Perkin Elmer (Waltham, MA, USA). This analysis followed the digestion of samples in a mixture of nitric acid (HNO_3_) and hydrochloric acid (HCl) at a ratio of 3:1 (*v*/*v*), utilizing a Milestone Ethos Up microwave digestion system (Sorisole, Italy).

### 2.12. Statistical Analysis

Results are provided as mean ± standard deviation (SD). Statistical analysis was conducted to compare biomass concentration, residual oil concentration, intracellular lipid content, and crude protein content across the semi-continuous cultures of *Y. lipolytica*. Each bioreactor variant was performed in two independent replicates, and each sample was analyzed in triplicate. Differences between means were assessed using one-way ANOVA, followed by Tukey’s post hoc test for multiple comparisons, with STATISTICA 13.1 (StatSoft, Kraków, Poland). Significance was set at *p* < 0.05.

## 3. Results

### 3.1. Single-Cell Oil Synthesis in Semi-Continuous Cultures of Y. lipolytica

**Figure 2:**
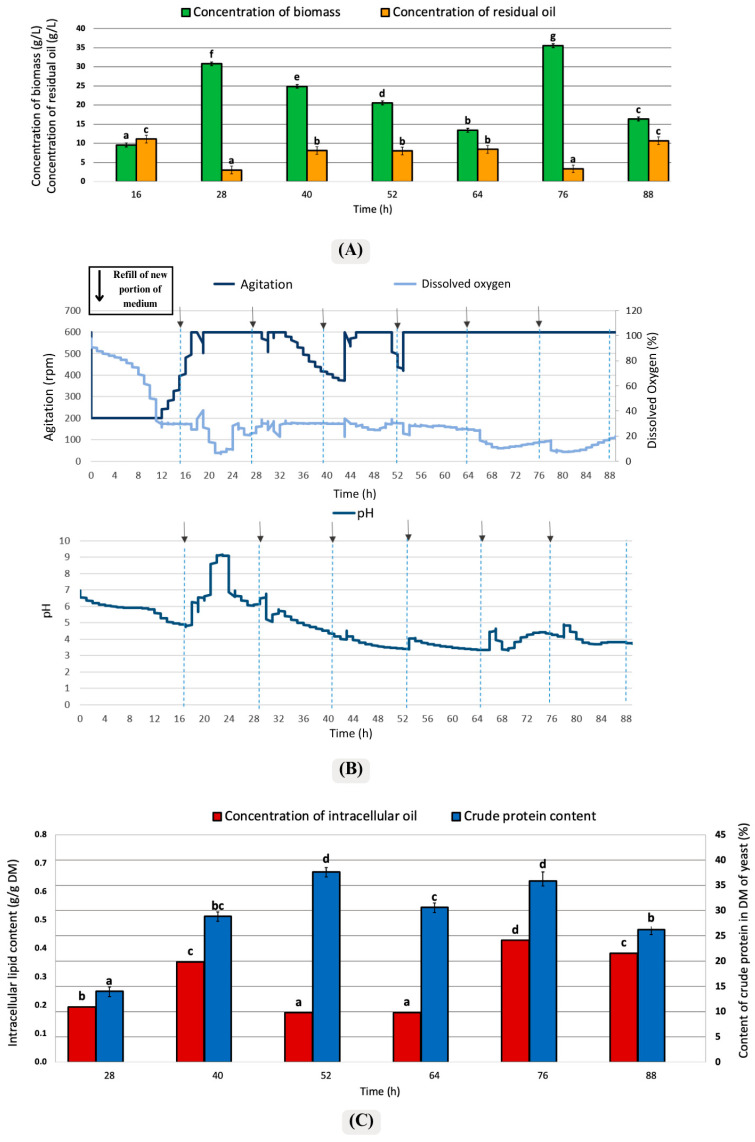
Changes in culture parameters in the variant of medium replacement frequency of 12 h: (**A**) Concentration of biomass yield and residual oil, (**B**) Agitation, dissolved oxygen, and pH changes, and (**C**) Intracellular lipid content and crude protein content. Error bars represent the standard deviation (SD). The values with the same lowercase letters (a–g) did not differ significantly (α = 0.05). The arrows (↓) represented the addition of a fresh medium portion.

In a series of laboratory experiments, the yeast strain *Y. lipolytica* KKP 379 was evaluated for its growth potential and lipid accumulation capabilities when cultured in media with waste post-frying rapeseed oil as a carbon source. Three Semi-Continuous cultures were conducted in a laboratory bioreactor, with three different frequencies of medium replacement. The results for the first culture, i.e., 12 h intervals, are presented in Figure 2.

According to Figure 2A, after the addition of the medium solution at the 16th hour, microorganisms rapidly consumed the available nutrients in the environment, resulting in a significant reduction in residual oil levels by the 28th hour. Moreover, a high biomass (30.78 g/L) and low residual oil content (3 g/L) were observed at the 28th hour. According to Figure 2B, the agitation rate increased at the 16th, 40th, and 52nd hours. Subsequently, the stable agitation rate observed was high (600 rpm) at the 76th and 88th hours, and yeasts were still in the logarithmic phase. The dissolved oxygen level increased after the medium solution was added at the 16th hour. After the 20th hour, the dissolved oxygen level started to decrease. The aerobic *Y. lipolytica* yeast in the bioreactor consumed the oxygen necessary for its metabolic activities. When the medium was added again at the 28th hour, it was observed that the oxygen level was sufficient, leading to a slight decrease in the agitation rate. Metabolic activities of the yeast were not very high at that point, and the dissolved oxygen level remained above 20%.

Similarly, during the medium additions at the 40th and 52nd hours, the agitation rate decreased while the dissolved oxygen level remained stable. In Figure 2B, after the addition of the medium solution at the 16th hour, the pH increased up to 9. After 52 h, fluctuations in pH became less pronounced as the metabolic activities of the yeast stabilized. At 76 h, the pH level remained relatively stable, indicating that the metabolic activities of the yeast had become more consistent and yeast cells were in the stationary phase.

According to Figure 2C, intracellular lipid production was relatively high after medium addition at the 40th, 76th, and 88th hours. The intracellular lipid content ranged from 0.19 to 0.35 g/g DM between 28 h and 40 h. A high intracellular lipid level was observed at the 40th hour, while similarly elevated values were maintained at the 76th and 88th hours. These results indicate that lipid accumulation was sustained during the later stages of cultivation under the tested conditions.

The crude protein content in the biomass reaches 37% at the 52nd hour and decreases slightly to 35% at the 76th hour. Particularly at the 52nd hour, which is 37% of crude protein in biomass, despite its high content, the lipid produced by the cells is low at approximately 20%. Lipid production generally increases when the carbon source is abundant, but the nitrogen source is limited. In this study, ex novo lipid accumulation should also be considered, since external fatty acids present in the medium may be directly taken up and incorporated into cellular storage lipids. In this case, the low lipid production may be associated with less favorable metabolic conditions for lipid accumulation rather than with an absolute limitation of the carbon source in the medium.

The culture was in a stable and unchanged form from 52 h of culture, i.e., from the fourth exchange of medium, although high constant intracellular fat production was obtained by d 72 h, i.e., from the sixth exchange of medium. The stability of the culture could be observed based on agitation speed and constant pH.

**Figure 3:**
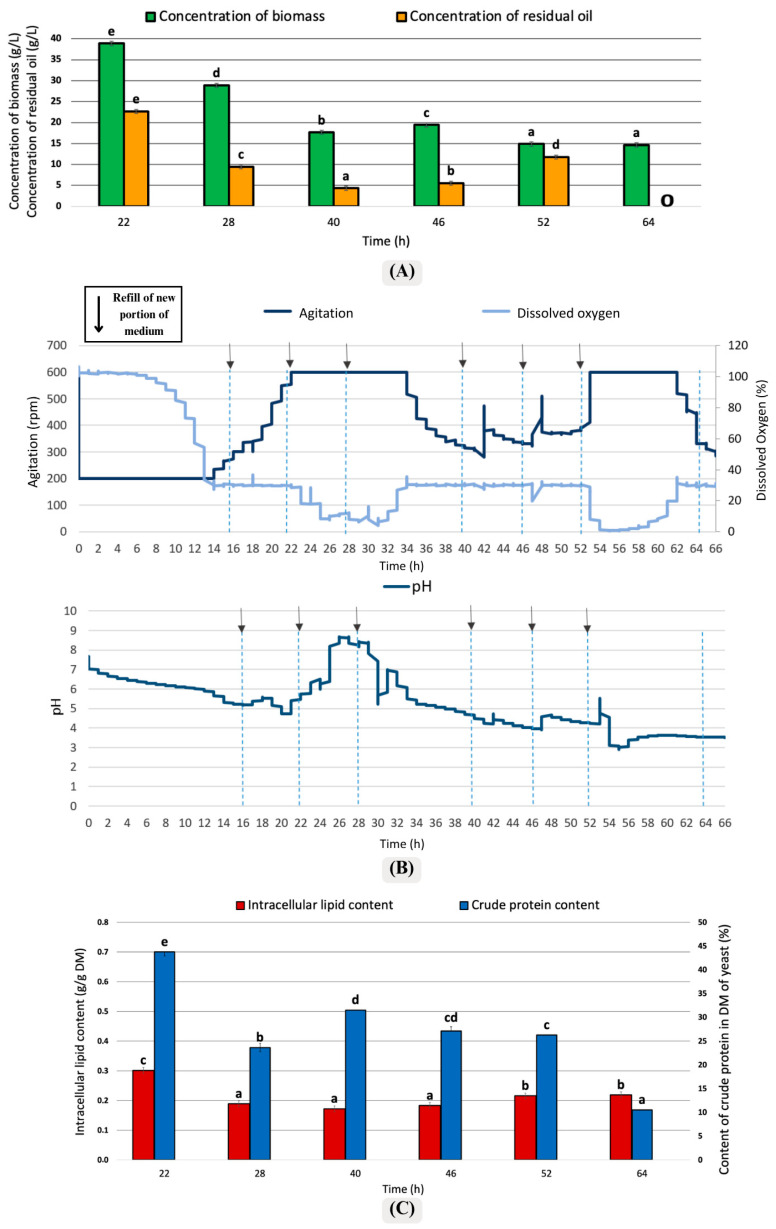
Changes in culture parameters in the variant of medium replacement in 6 h–6 h–12 h frequencies: (**A**) Concentration of biomass yield and residual oil; (**B**) Agitation and pH changes, and (**C**) Intracellular lipid content and crude protein content. Error bars represent the standard deviation (SD). The values with the same lowercase letters (a–e) did not differ significantly (α = 0.05). The arrows (↓) represented the addition of fresh medium.

The second distinct period for refilling the medium was a frequency of 6–6–12 h per day intervals (Figure 3). According to Figure 3A, the biomass ratio is high at the 22nd (38 g/L) and 28th (28 g/L) hours and remained stable during the subsequent hours. The cells continued their metabolic activity; they did not completely consume the carbon sources in the environment and, therefore, could not increase lipid production. The cells were actively synthesizing lipids during this time. In Figure 3B, examining the dissolved oxygen levels shows that after the medium solution was added at the 16th hour, a significant increase was observed in the graph, with a temporary rise in the dissolved oxygen level. As the metabolic activities of *Y. lipolytica* in the environment increased, the dissolved oxygen level decreased again and stabilized, supporting the idea that a high oxygen level is necessary to produce lipids in yeast cells. Since oil was used as the substrate, the cultivation medium represented a two-phase system consisting of an aqueous phase and an oil phase. Therefore, the dissolved oxygen profile may also have been influenced by differences in oxygen transfer and solubility between these phases.

After the addition of medium at the 40th and 46th hours, agitation increased rapidly, while dissolved oxygen showed only minor changes. Following the final medium addition at the 52nd hour, a sharp decrease in dissolved oxygen was accompanied by a rapid increase in agitation, indicating a rapid response of the culture to renewed nutrient availability. According to Figure 3C, intracellular lipid content decreased after the 28th hour. In parallel, the pH increased toward the 26th hour and then slightly decreased after the medium addition at the 40th hour, suggesting a shift in culture conditions during this stage.

In Figure 3C, the crude protein content and intracellular lipid ratio are highest at the 22nd hour. At the 46th and 52nd hours, there is no statistically significant difference in crude protein content.

The culture was unstable under the 6–6–12 h medium exchange pattern, as the short intervals did not allow yeast cells sufficient time to consume nutrients before the next exchange. Although biomass remained high, lipid production was limited, with intracellular lipid levels peaking at 22 h (0.3 g/g) before declining.

**Figure 4:**
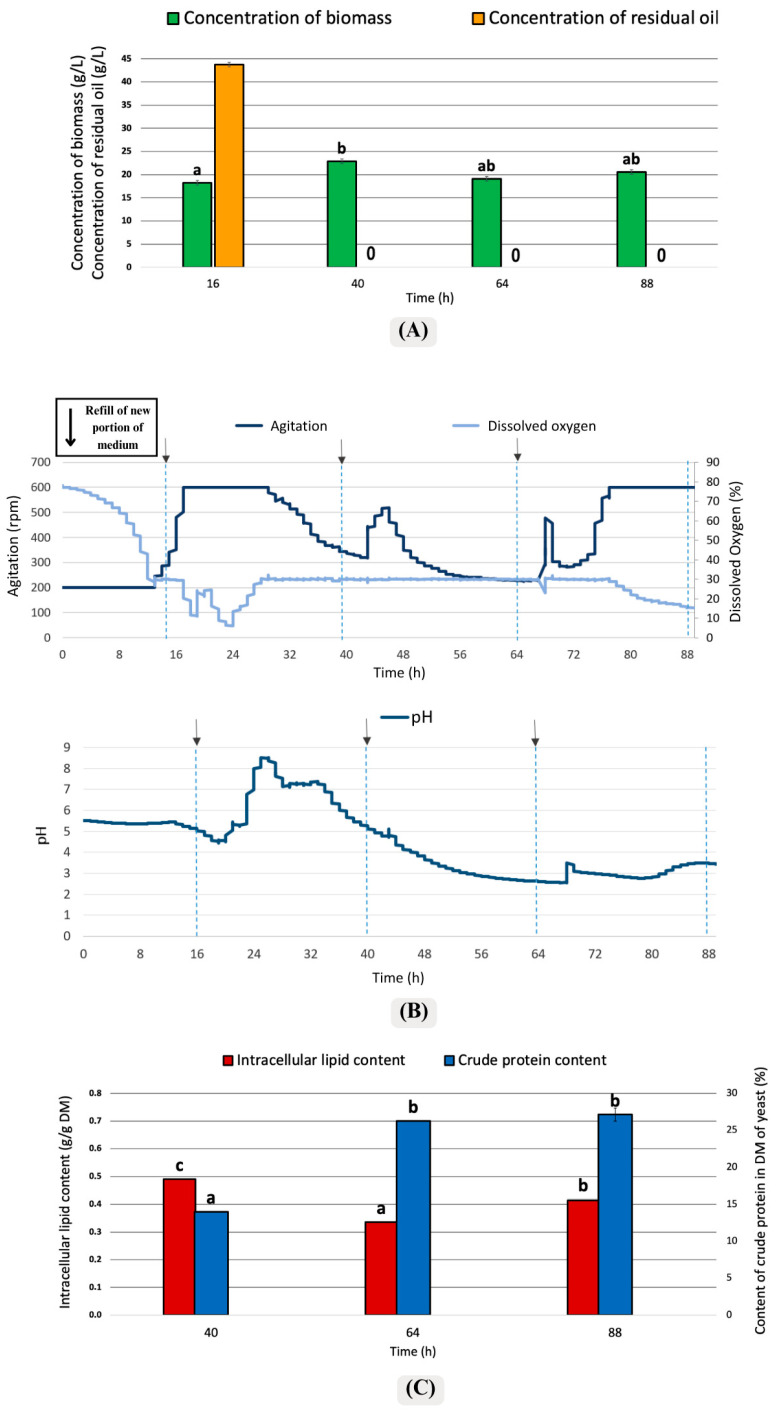
Changes in culture parameters in the variant of medium replacement once a 24 h: (**A**) Concentration of biomass yield and residual oil; (**B**) Agitation and pH changes; and (**C**) Intracellular lipid content and crude protein content. Error bars represent the standard deviation (SD). The values with the same lowercase letters (a–c) did not differ significantly (α = 0.05). The arrows (↓) represented the addition of fresh medium.

The third mode for refilling the medium was a frequency of 24 h intervals (Figure 4). According to Figure 4A, 40 g/L of residual oil is observed at the end of the first 16 h. This indicates that the yeast cells did not consume the oil provided as a carbon source, leading to the remaining oil in the medium. Then, during the culture, over 24 h intervals, the microorganisms consumed all the carbon source added to the medium solution.

In Figure 4B, dissolved oxygen decreased below 20% after the 16th hour, accompanied by an increase in agitation speed to 600 rpm. After the 24th hour, the pH increased markedly, approaching 9. Following the 40th and 64th hours, the pH decreased and then stabilized at approximately 3. According to Figure 4C, intracellular lipid production remained at 30% or higher during the later stages of cultivation. In addition, no statistically significant difference in crude protein content was observed between the 64th and 88th hours, with crude protein remaining at approximately 26%. At the 40th hour, the crude protein proportion in dry mass was lower (14%), while intracellular lipid synthesis was high.

Notably, 24 h intervals were effective because they provided sufficient time for yeast cells to fully consume the available nutrients before the next medium exchange. In shorter intervals (e.g., 6–6–12 h), frequent medium replacement disrupted metabolic stability, preventing optimal lipid synthesis. With 24 h intervals, yeast cells could complete their nutrient uptake, transition into the lipid production phase, and maintain a stable metabolic state, leading to higher intracellular lipid accumulation and improved culture efficiency.

The results from Table 2 regarding the kinetic parameters of *Y. lipolytica* KKP 379 in semi-continuous cultures provide insights into biomass production and lipid accumulation across distinct semi-continuous culture setups. The data indicate that different feeding strategies significantly impact both biomass and lipid production in *Y. lipolytica* KKP 379 cultures. The 22nd, 40th, and 76th hours correspond to the time points at which biomass concentrations were highest. Cultures with longer feeding times tend to yield higher biomass concentrations, while variations in lipid yield suggest that optimizing feeding strategies could enhance overall lipid production efficiency. In the second culture, where the medium refill was more frequent, lipid accumulation was less efficient compared to cultures 1 and 3. After 22 h, the concentration of cellular lipids (L_max_) reached 11.67 g/L, and ended at 3.23 g/L. However, this culture exhibited the highest biomass yield, achieving 38.89 g/L. Culture 3 exhibits a more consistent performance, achieving favorable yields throughout the cultivation interval, with the highest recorded storage lipid yields per biomass of 0.49 g/g and 0.41 g/g, respectively, after 40 and 88 h of culture. The drop in biomass content over time in the cultures can be attributed to oil production and oxygenation levels; as oxygen becomes limited, biomass production decreases. The reduction in (L_max_) could be due to nutrient imbalance and substrate depletion. In the second culture, where feeding was more frequent, the (Y_L/X_) ratio decreased, indicating that too frequent substrate changes can reduce efficiency. In conclusion, the frequency of substrate changes should be optimized to achieve the best biomass and lipid production. Too frequent changes may disrupt the culture, leading to inefficiencies and reducing long-term profitability.

### 3.2. Determination of Fatty Acids Composition

The profiles of intracellular fatty acids produced as a result of experiments conducted in a semi-continuous culture bioreactor during three different frequencies and the post-frying waste oil used as the substrate are presented in Table 3. The fatty acid profile obtained through gas chromatography demonstrates which fatty acids were produced and how their proportions changed during the cells’ lipid synthesis process. It is generally observed that the proportion of saturated fatty acids is low, though it increases at specific times.

According to Table 3A, the fatty acid composition exhibits a relatively stable structure during 12 h intervals. The proportion of saturated fatty acids (SFA) ranges from 5.33% to 7.73%, remaining at low levels. In contrast, the proportion of unsaturated fatty acids (UFA) is high, ranging from 91.99% to 94.65%, with the highest value observed at the 88th hour. Among these, monounsaturated fatty acids (MUFA) peak at 71.76% at the 88th hour, among with the polyunsaturated fatty acids (PUFA) with 29.98% at the 28th hour. The fatty acid with the highest share is C18:1 (oleic acid) at 68.78%, followed by C18:2 (linoleic acid).

Table 3B represents 6–6–12 h intervals and reveals more dynamic changes in the fatty acid composition. Saturated fatty acids (SFA) fluctuate between 5.14% and 10.30%. Meanwhile, unsaturated fatty acids (UFA) maintain a range of 89.69% to 94.90%, with MUFA (64.34% to 84.12%) being dominant. In this table, C18:1 has the highest proportion, ranging from 63.30% to 68.93%. PUFA shows a notably high value of 29.98% at the 22nd hour. Similarly, the most abundant fatty acid is C18:1, peaking at 81.24%, while C18:2 (linoleic acid) is absent at the 64th hour, a noteworthy observation.

Table 3C covers 24 h frequencies and demonstrates some differences in fatty acid composition compared to the other two tables. The SFA proportion ranges from 6.43% to 8.60%, while the UFA proportion ranges from 91.40% to 93.57%, peaking at the 40th hour. Likewise, MUFA reaches its highest level of 93.57% at the 40th hour. The PUFA proportion decreases steadily from 27.93% to 20.78%. Once again, the fatty acid with the highest share is C18:1, ranging between 62.96% and 68.93%.

Overall, the microbial oils obtained from all three cultivation strategies were characterized by a predominance of unsaturated fatty acids over saturated fatty acids, with total UFA content averaging approximately 90%. In all cultures, both MUFA and PUFA fractions remained high, and C18:1 (oleic acid) and C18:2 (linoleic acid) were consistently detected at considerable proportions.

The fatty acid composition of the intracellular lipids clearly reflected the fatty acid profile of the post-frying waste oil used as the carbon source. The substrate was mainly composed of oleic acid (C18:1; 61.73%), followed by linoleic acid (C18:2; 19.26%) and linolenic acid (C18:3; 7.51%). Across all cultivation variants, the microbial lipids were similarly composed, with C18 fatty acids predominating. Notably, oleic acid remained the dominant fatty acid in yeast lipids, ranging from 62.71% to 81.24%, with linoleic and linolenic acids also consistently detected but usually at lower levels than in the substrate. This indicates that the fatty acid profile of the accumulated lipids was largely influenced by the composition of the waste frying oil.

### 3.3. Freeze-Dried Supernatant

The data obtained from *Y. lipolytica* cultures highlight the potential of this microorganism for various biotechnological applications. Moreover, analysis of post-culture waste (supernatant) revealed its interesting content (Table 4 and Table 5). With a calorific value of 10.4335 kJ/g, it could be a future alternative feedstock for bioenergy production (Table 4). The residual reducing sugar content (14.39 mg glucose equivalents/g DW) could be utilized in secondary processes. The high ash content (30.27%) and sulfur level (3.58%) indicate the need for additional processing to mitigate their potential effects on downstream applications and emissions. Elemental analysis reveals that trace elements such as iron (2.79 mg/kg) and zinc (2.00 mg/kg) contribute to its biological and industrial utility, while the presence of toxic elements like lead (0.010 mg/kg) and arsenic (0.029 mg/kg) at safe levels (Table 5). Furthermore, elements critical for metabolic processes, such as phosphorus (430 mg/kg) and potassium (428 mg/kg), are present in high amounts, suggesting the potential use of biomass as a nutrient-rich biofertilizer or biostimulant.

## 4. Discussion

### 4.1. Concentration of Biomass Yield, Residual Oils, and Concentration of Intracellular Lipids

In experiments conducted in semi-continuous culture, different frequencies of medium feeding were studied to increase biomass yield and intracellular lipid production. From an industrial and bioprocessing perspective, continuous and semi-continuous cultivation strategies have already been explored for lipid-related processes in *Y. lipolytica*, including single-stage continuous culture, stepwise continuous fed-batch, chemostat, and semicontinuous cell-recycle systems [33,34,35,36]. These cultivation modes are appealing because they can provide more controlled, reproducible operating conditions, maintain a more stable physiological state, reduce downtime associated with repeated reactor start-up, and extend the productive phase of the process [36]. At the same time, their performance heavily depends on dilution rate or medium-exchange frequency, nutrient-limitation strategy, oxygen availability, and the ability to sustain stable process conditions over long periods [36]. In the case of *Y. lipolytica*, this point is especially important because lipid accumulation is not simply linked to rapid biomass growth, but is closely related to the metabolic state of the cells under appropriate nutrient and carbon supply conditions [37,38]. The feasibility of such strategies in *Y. lipolytica* has already been demonstrated in various process configurations. Papanikolaou and Aggelis [38] showed that *Y. lipolytica* could accumulate lipids in a single-stage continuous culture using industrial glycerol, laying an early foundation for the use of continuous cultivation with this yeast [38]. Later, Rakicka et al. [34] demonstrated lipid production by an engineered *Y. lipolytica* strain under stepwise continuous fed-batch and chemostat conditions using crude glycerol and molasses, confirming that long-term controlled cultivation can support both high biomass growth and significant intracellular lipid accumulation [34]. Xu et al. [35] further advanced semicontinuous lipid production by combining cell recycling with dynamic carbon and nitrogen management during acetate feeding, achieving very high lipid titers and productivity while maintaining complete substrate utilization [35]. More recently, Tsirigka et al. [39] showed that different fermentation modes, including a semi-continuous membrane bioreactor approach using crude glycerol, can improve process selectivity and suppress citric acid secretion, further supporting the potential of semi-continuous *Y. lipolytica* systems for intensified cultivation [39]. In this context, the present study evaluates a simpler semi-continuous strategy based on periodic partial medium replacement rather than full chemostat operation or membrane-assisted cell retention. This approach still preserves key advantages of semi-continuous cultivation, including regular nutrient renewal, extending the productive phase, and maintaining an active biomass population, while avoiding the additional technical complexity associated with continuous-feed control loops or membrane modules [35,36]. At the same time, the results of this work show that the frequency of medium exchange strongly influences the balance between biomass formation, intracellular lipid accumulation, and process stability. This observation aligns with broader literature, which indicates that semi-continuous cultivation in *Y. lipolytica* is not governed by a single universally optimal regime, but rather by the need to adjust process parameters based on the specific goal, whether that is maximal biomass production, high lipid accumulation, or more stable long-term culture performance [34,36,39].

It was observed that as the medium solution was added, microorganisms consumed the carbon source and increased intracellular lipid accumulation. The observed lipid accumulation may be explained by the oleaginous metabolism of *Y. lipolytica,* which can utilize post-frying oil as an external carbon source, take up and assimilate fatty acids from the medium, and convert them into intracellularly stored lipids. In particular, the intracellular lipid synthesis capacity of *Y. lipolytica* was generally high and provided lipid accumulation exceeding 30%. In a similar study conducted by Karamerou et al. [40] with *Rhodotorula glutinis* using the semi-continuous method, a feeding strategy with medium was applied. In this study, in which glycerol was used as the carbon source, it was determined that controlled glycerol additions increased cell growth and produced intracellular oil [40]. The maximum intracellular lipid ratio obtained for *R. glutinis* species was 34.62%, above the lipid accumulation observed in the study with *Y. lipolytica.* These results suggest that this yeast species can accumulate higher lipid levels under these conditions. The fact that the intracellular lipid ratio approached 50% at the 40th hour, as shown in Figure 3C, indicates the high performance of this yeast in microbial oil production.

Regarding residual oil, this study and the study by Karamerou et al. [41] showed that microorganisms consumed the carbon source. In the experiment with *Y. lipolytica*, biomass yield was increased as the amount of oil remaining in the medium decreased; this situation was similar to that observed in the study on *R. glutinis* [40]. In Karamerou’s study [40], higher biomass yield was obtained with the continuous feeding strategy; this supported that it was a critical factor affecting the amount of biomass. Previous studies have shown that batch and fed-batch modes can also support metabolite production in yeasts. However, these strategies were not evaluated in the present study, which focused exclusively on semi-continuous cultivation. The fed-batch feeding method has been shown to enhance the oil production capacity of microorganisms. In a study by Wierzchowska et al. [41], *Y. lipolytica* cultures were conducted in both batch and fed-batch modes using molasses hydrolysate and post-frying rapeseed oil as carbon sources. The results indicated that the fed-batch cultures maintained cellular lipid content at high levels (30.75–31.73%) for 50 h, with a maximum yield of 37.50% [41].

According to the study conducted with *R. glutinis,* the cellular lipid ratio obtained with *Y. lipolytica* yeast was higher. It was determined that the oil production capacity of microorganisms fed with a fed-batch culture was higher than that of the batch culture method. The high efficiency provided by *Y. lipolytica* in intracellular lipid accumulation, especially the lipid synthesis of over 30% at most hours, reveals a more effective biomass and lipid accumulation than the results obtained by Karamerou et al. with *R. glutinis* [42].

The study by Zhang et al. [43] compared (+)-borneol production using the fed-batch feeding strategy with the batch method in *Saccharomyces cerevisiae*. The findings indicate that the fed-batch method, through controlled nutrient supplementation, optimizes cell metabolism and enables higher (+)-borneol production [43]. Notably, the production level reached 753 mg/L, demonstrating that fed-batch fermentation significantly enhances the microorganism’s metabolite production compared to the batch method [43]. According to this result, the semi-continuous and fed-batch culturing methods are more suitable for microbial lipid and other secondary metabolite production, as they promote cell growth and metabolic efficiency.

### 4.2. Agitation, Dissolved Oxygen, and pH Changes

In a study on microbial oil production using *R. glutinis*, agitation speed and dissolved oxygen ratios were investigated [44]. Low dissolved oxygen levels (15% to 35%) were found to enhance lipid accumulation, with lipid content reaching 63.4% in low DO (Dissolved Oxygen) conditions compared to 47.3% in high DO (50% to 70%). Conversely, high DO conditions facilitated faster cell growth and greater biomass production, although reduced lipid accumulation per unit biomass. Agitation speed, which influences dissolved oxygen levels by enhancing oxygen transfer, was shown to promote cell growth at higher speeds while suppressing lipid accumulation [44]. *Y. lipolytica* is an aerobic microorganism, requiring oxygen for its metabolic activity. According to Figure 3B, the increase in dissolved oxygen levels in the environment supports the metabolic activities of *Y. lipolytica* microorganisms, thereby supporting higher lipid accumulation. While the agitation speed increased, aiming to restore oxygen levels in the environment. This indicates that *Y. lipolytica* requires high oxygen levels for both growth and lipid production. Furthermore, after the 24th hour, the increase in dissolved oxygen levels in the medium correlates with intracellular lipid production reaching approximately 50%, further supporting this observation. It was also an interesting observation that the metabolic intensity of cells correlated with culture feeding and oxygen demand [44].

The pH charts presented in Figure 2B, Figure 3B, and Figure 4B demonstrate a notable rise in pH to 9 during a specific time interval in all three cases. This observation aligns with findings from other studies, where environments with limited carbon sources exhibited an increase in pH upon the external addition of nitrogen-containing compounds, such as amino acids. These compounds are metabolized and utilized for cell growth, leading to the production of nitrogen metabolism byproducts, such as ammonia, which subsequently result in the alkalization of the environment [45].

### 4.3. Crude Protein Content

According to Figure 2C, the crude protein ratio approached 35% at the 52nd and 76th hours, and no statistically significant difference was found between these intervals. In Figure 3C, the amount of protein in the biomass obtained after the 22nd hour approached 45% and reached the highest level. Figure 4C, the amount of crude protein in the biomass remained around 27%. It was observed that the feeding model performed with the semi-continuous method affected protein production of *Y. lipolytica* satisfactorily. In another study conducted by Jach et al. [46], conducted with *Y. lipolytica*, protein production was increased by optimizing parameters such as pH and temperature and using a standard YPD (containing glucose, peptone, and yeast extract) medium [46]. The protein concentration, which was 3.65% under standard conditions, increased by 44% and reached 8.28% when biofuel waste was used. According to results from Jach and her coworkers, the highest protein amount was measured as 11.57% at 30 °C and pH 5.0 [46]. According to the current study and the studies from Jach et al. [46], a satisfactory protein amount was determined in the biomass of *Y. lipolytica*. It was found that the chosen culture mode significantly enhanced protein production. The frequent addition of nutrients boosted the yeast’s capacity for protein production, resulting in higher protein levels in the biomass compared to the other study conducted using the batch fermentation method.

Furthermore, the research by Juszczyk et al. [47] utilized a batch culture method using *Y. lipolytica* yeast. In this study, glycerol served as the raw material, yielding a protein ratio in the resulting biomass of 40–45% [47].

Both methods offer strategies to increase the protein production capacity of *Y. lipolytica*. While the fed-batch method allows higher protein rates to be obtained with dynamic feeding depending on the intervals, the use of alternative nutrient sources, such as biofuel waste, provides both environmental and economic benefits. Protein in biomass is both an alternative source for vegans and supports sustainability because it is obtained by using waste.

Recent studies by Pobiega et al. [48] highlight the extensive use of microbial proteins from fungi as alternative protein sources in the food industry *Fusarium venenatum*-derived mycoproteins, such as Quorn™, are commonly utilized as meat substitutes due to their high protein content and meat-like texture. Moreover, microbial proteins from *S. cerevisiae* and *Candida utilis* serve as functional ingredients in high-protein products, including protein bars, shakes, and dairy alternatives [48]. These proteins also function as natural flavor enhancers, contributing to umami taste and improving the nutritional profile of foods. Based on this and previous research, single-cell proteins (SCPs) are increasingly integrated into sustainable food production systems by utilizing agricultural and food industry waste as fermentation substrates. This process not only aids in waste valorization but also reduces the environmental footprint of protein production [48].

Microbial protein sources such as *Y. lipolytica* and *S. cerevisiae* are widely used in animal feed to enhance nutritional value. These yeasts provide essential amino acids, B vitamins, and bioactive compounds that support animal health and growth. Pobiega et al. [48] also indicate that SCPs have promising applications in biotechnology and pharmaceuticals, particularly in enzyme production and probiotic formulations [48].

### 4.4. Composition of Fatty Acids

The fatty acids found in biomass were examined as a result of the experiment carried out in 3 different frequencies (A, B and C). According to Table 3A, *Y. lipolytica* yeast synthesized C16:0, C18:0, C18:1, C18:2, C20:0 fatty acids mostly. Generally, the UFA rate obtained was higher across all frequencies (~92%). The MUFA ratio obtained in all frequencies was higher than the PUFA ratio (~68%). According to another study by Fabiszewska et al. [29] when the fatty acids produced by *Y. lipolytica* were examined, the most common ones were determined to be C16:0, C18:0, C18:1, C18:2 and C18:3. In the current study, the differences between the substrate and microbial lipids indicate that the process was not solely based on the direct incorporation of external fatty acids. The observed reduction in polyunsaturated fatty acids and the increase in monounsaturated fatty acids suggest that *Y. lipolytica* not only absorbed hydrophobic substrate components but also modified them through intracellular metabolism, including elongation, desaturation, and redistribution between structural and storage lipids [37,38]. This behavior aligns with the known physiology of *Y. lipolytica*, which can utilize fats, oils, and free fatty acids ex novo and reshape its intracellular lipid profile in response to substrate composition and cultivation conditions [38]. Previous studies have similarly shown that, in *Y. lipolytica*, the major fatty acids generally remain within the C16–C18 range and that variations in fatty acid composition are more often driven by the type of carbon source rather than by the culture mode itself [34,37].

The study of Juszczyk et al. [47] with *Y. lipolytica* also found that when the fatty acid composition was examined, the most common fatty acids were C16:0, C18:1, and 18:2 fatty acids [38]. Another study, which was conducted by Carsanba and colleagues [49] on *Y. lipolytica*, the microbial oil obtained was found to contain myristic (C14:0), palmitic (C16:0), palmitoleic (C16:1), stearic (C18:0), oleic (C18:1), and linoleic (C18:2) acids [49]. Bettencourt et al. [50] investigation into oleaginous (lipid-producing) microorganisms revealed that *Apiotrichum brassicae* and *Pichia kudriavzevii* produced high amounts of lipids, containing more than 40% oleic acid (C18:1), 20% palmitic acid (C16:0), and 20% stearic acid (C18:0). In contrast, *Candida tropicalis* and *Metschnikowia pulcherrima* exhibited a distinct fatty acid profile, consisting of 60% oleic acid (C18:1), 10% palmitoleic acid (C16:1), and 5% linoleic acid (C18:2). The results highlight significant variations in both the lipid accumulation capacity and fatty acid composition among different yeast species [50].

Palmitic acid was produced by the cells at all times. Moreover, the cells synthesized 16:1 (palmitoleic acid) and 18:0 (stearic acid) during most time frequencies. Oleic acid (C18:1) was found in most frequencies and across all tables. Linoleic acid (C18:2) also consistently appeared at various time intervals and with a share of up to 25%. The unsaturated fatty acids, which are produced in large amounts by cells across all 3 tables, have numerous health benefits, including protecting heart health, reducing inflammation in the body, and supporting brain function. The predominance of unsaturated fatty acids, especially oleic acid (C18:1) and linoleic acid (C18:2), clearly highlights the biotechnological and nutritional value of the produced microbial oil. Owing to its fatty acid composition, which closely resembles that of conventional vegetable oils, this microbial oil represents a promising sustainable alternative lipid source. Its composition suggests strong potential for food applications and supports its possible use as a substitute, at least in part, for plant-derived oils in future formulations. Monounsaturated fatty acids, known for increasing HDL cholesterol, play a role in reducing cardiovascular diseases. Also have anti-inflammatory properties, which contribute to reducing the risk of chronic conditions such as heart disease and diabetes. Additionally, MUFAs support blood sugar regulation and enhance insulin sensitivity, making them beneficial for individuals with type 2 diabetes [51].

### 4.5. Freeze-Dried Supernatant

In the microbial lipid production process using *Y. lipolytica*, the high-volume supernatant was freeze-dried, and the dried product was subjected to chemical and elemental analyses. The supernatant exhibited a significant calorific value and high concentrations of essential elements such as phosphorus and potassium. It seems that it might be useful for future applications in bioenergy and biofertilizer production. Similarly, another study utilized freeze-dried *Y. lipolytica* biomass to extract lipids and analyze its fatty acid profile, demonstrating that freeze-drying effectively preserved biomass integrity. This aligns with our results, where freeze-drying played a crucial role in analyzing the broader chemical properties of the supernatant. In a related study, de França Bettencourt et al. [52] investigated *Paenibacillus polymyxa*, revealing that this bacterium can ferment tryptophan to produce auxins such as indole-3-acetic acid (IAA) [52]. Additionally, its supernatant was found to play a dual role in both the synthesis and stabilization of nanoparticles. Acting as a reducing and capping agent, the supernatant facilitated the formation of iron (FeOx) and manganese (MnOx) nanoparticles, which demonstrated superior plant growth-promoting effects. The bioactive compounds present in the supernatant not only enhanced nanoparticle stability but also contributed to improved plant growth, suggesting a sustainable alternative to conventional fertilizers.

Further research by Mao et al. [53] explored the potential of lactic acid bacteria (LAB) supernatants obtained from both planktonic and biofilm cultures [53]. These supernatants enhanced soil microflora by promoting the growth of beneficial microorganisms. Additionally, their bioactive metabolites accelerated plant growth and reduced reliance on chemical fertilizers, highlighting their potential application in sustainable agriculture.

From an industrial perspective, semi-continuous cultivation may offer practical advantages over batch operation by enabling longer production campaigns, tighter process control, and a more stable physiological state of the culture, thereby enhancing reproducibility and volumetric productivity when the operating regime is properly chosen [36]. In oleaginous yeast systems, semi-continuous processing has already been demonstrated at pilot scale, where extended cultivation supported a relatively stable lipid production rate and fatty acid profile during long-term operation, emphasizing the importance of this strategy for scale-up [54]. At the same time, for *Y. lipolytica,* continuous and semi-continuous modes still require careful optimization, as lipid accumulation is often most strongly linked to nutrient-limited post-growth conditions, and batch/fed-batch processes remain the more established methods for achieving high intracellular lipid accumulation [11,36]. Using post-frying waste oil as the primary carbon source further enhances the industrial relevance of the process [55,56]. Low-cost waste and by-product streams are widely recognized as key factors for improving the economic viability of microbial oil production, and *Y. lipolytica* has consistently been identified as an effective biocatalyst for converting waste cooking oils and other hydrophobic residues [11,56]. In this context, the process combines lipid production with waste management, making it attractive from both circular-economy and resource-efficiency perspectives. Nevertheless, industrial implementation depends not only on fermentation performance but also on downstream processing and overall process economics. Reviews and techno-economic analyses consistently identify harvesting, drying, cell disruption, lipid extraction, solvent recovery, and energy consumption as major bottlenecks for intracellular oil production [57]. Modeling studies further show that the minimum selling price is highly sensitive to electricity demand, lipid-recovery strategy, process scale, and the possibility of marketing whole biomass or additional coproducts [57,58].

Overall, supernatants derived from various microorganisms hold significant promise for diverse applications, including bioenergy, agriculture, and biotechnology. For instance, a study on *Streptococcus alactolyticus* examined its supernatant’s antioxidant activity and metabolomic profile, identifying key antioxidant compounds [59]. Due to these properties, it is a promising probiotic with potential applications in supporting animal health and feed supplements [60,61].

## 5. Conclusions

This study demonstrated that *Y. lipolytica* can effectively produce microbial lipids under semi-continuous cultivation using waste post-frying oil as a substrate. Among the tested feeding strategies, the 24 h interval showed the most favorable overall performance. However, the present findings should be interpreted as a comparison among the tested semi-continuous feeding strategies. The results also showed that the biomass contained a considerable crude protein fraction, indicating the additional nutritional potential of the produced microbial biomass. Moreover, the microbial oils obtained from all three cultivation strategies were characterized by a predominance of unsaturated fatty acids, particularly C18:1 (oleic acid) and C18:2 (linoleic acid). In addition, the freeze-dried post-culture supernatant showed potential for further utilization as a value-added by-product. Overall, these findings support the potential of combining microbial lipid production with a sustainability-oriented approach based on waste-derived substrates and by-product valorization.

## Figures and Tables

**Table 1:** Characteristic fatty acids produced by selected oleaginous microorganisms.

Microorganism	Characteristic Fatty Acids	References
*Chlorella* sp.	ALA (C18:3), EPA (C20:5), DHA (C22:6)	[14]
*Mortierella alpina*	High ARA (C20:4, omega-6)	[15]
*Schizochytrium* sp.	High DHA (C22:6)	[16]
*Crypthecodinium cohnii*	DHA (C22:6)	[17]
*Thraustochytrium* sp.	DHA (C22:6), EPA (C20:5)	[18]
*Y. lipolytica*	Oleic acid (C18:1, omega-9), Linoleic acid (C18:2, omega-6)	[19]

Abbreviations: ALA—α-linolenic acid (C18:3, omega-3), EPA—eicosapentaenoic acid (C20:5, omega-3), DHA—docosahexaenoic acid (C22:6, omega-3), ARA—arachidonic acid (C20:4, omega-6).

**Table 2:** Kinetic parameters of *Y. lipolytica* KKP 379 semi-continuous cultures.

No. Culture	Frequency of Medium Replacement	T (h) *	X (g/L)	L_max_ (g/L)	Y_L/X_ (g/g)
1	12 h	76	35.56	15.29	0.43
		88	16.33	6.21	0.38
2	6–6–12 h	22	38.89	11.67	0.30
		64	14.67	3.23	0.22
3	24 h	40	22.89	11.22	0.49
		88	20.56	8.43	0.41

* Time (T), biomass concentration (X), concentration of cellular lipids (L_max_), storage lipids yield per biomass (Y_L/X_).

**Table 3:** Fatty acid composition (% of total identified fatty acids) of post-frying waste oil (WFO) and lipids extracted from *Y. lipolytica* biomass obtained under different medium replacement frequencies: (A) 12 h, (B) 6 h–6 h–12 h, and (C) 24 h, at selected cultivation times.

Fatty Acid	WFO	(A) 12 h Frequency							(B) 6–6–12 h Frequency						(C) 24 h Frequency		
		16 h	28 h	40 h	52 h	64 h	76 h	88 h	22 h	28 h	40 h	46 h	52 h	64 h	40 h	64 h	88 h
C14:0	0.15	-	0.23	0.13	0.16	0.14	0.15	0.11	-	0.64	0.47	-	0.18	0.11	-	-	-
C16:0	6.52	5.00	4.84	4.08	3.74	4.70	4.37	3.66	3.45	6.89	5.53	4.06	3.90	4.75	3.90	5.76	4.75
C16:1	0.34	0.28	2.39	4.60	3.51	2.07	0.11	0.61	0.58	0.46	0.44	3.21	1.88	1.00	1.73	2.74	0.92
C17:0	0.14	-	-	0.12	-	-	-	-	-	-	-	-	-	-	-	0.29	0.36
C17:1	0.08	-	-	0.14	0.12	-	-	-	-	-	-	-	-	-	0.10	-	-
C18:0	2.34	1.83	2.28	1.73	2.04	1.93	2.31	0.04	1.59	2.18	0.06	1.80	0.04	2.76	2.15	2.03	2.12
C18:1	61.73	64.99	62.90	63.75	64.43	64.64	66.37	68.78	62.71	63.30	66.27	65.22	67.61	81.24	62.96	65.54	68.93
C18:2	19.26	20.43	19.67	18.51	18.45	18.22	17.81	17.30	22.97	18.90	18.02	18.62	17.72	-	23.01	18.67	17.11
C18:3	7.51	5.44	6.29	5.67	5.77	6.14	5.82	5.99	7.01	5.82	6.09	5.63	5.89	7.61	4.92	3.54	3.67
C20:0	0.61	0.88	0.38	0.44	0.52	0.50	0.90	0.93	0.64	0.53	0.96	0.38	0.85	0.64	0.38	0.52	0.69
C20:1	0.97	1.16	1.02	0.69	1.12	1.28	1.88	1.93	1.05	1.21	1.96	1.07	1.78	1.88	0.85	0.91	1.45
C22:0	-	-	-	0.13	0.13	-	-	0.15	-	0.06	0.12	-	0.15	-	-	-	-
C22:1	-	-	-	-	-	-	-	0.04	-	-	0.02	-	0.02	-	-	-	-
C24:0	0.33	-	-	-	-	-	-	0.44	-	-	-	-	-	-	-	-	-
SFA	10.09	7.71	7.73	6.63	6.59	7.27	7.73	5.33	5.68	10.30	7.16	6.24	5.14	8.26	6.43	8.60	7.92
UFA	89.89	92.30	92.27	93.36	93.40	92.35	91.99	94.65	94.32	89.69	92.80	93.75	94.90	91.73	93.57	91.40	92.08
MUFA	63.12	66.43	66.31	69.18	69.18	67.99	68.36	71.36	64.34	64.97	68.69	69.50	71.29	84.12	65.64	69.19	71.30
PUFA	26.77	25.87	25.96	24.18	24.22	24.36	23.63	29.98	29.98	24.72	24.25	24.25	23.61	7.61	27.93	22.21	20.78

WFO—Post-frying waste oil; SFA—saturated fatty acids; UFA—unsaturated fatty acids; MUFA—monounsaturated fatty acids; PUFA—polyunsaturated fatty acids; “-”—not detected.

**Table 4:** Physicochemical parameters of freeze-dried supernatant from *Y. lipolytica* cultures.

Parameter	Unit	Value
Calorific Value	kJ/g	10.4335 ± 0.005
Lipid Content	g/g DW	0.37 ± 0.01
Reducing Sugar Content	mg glucose equivalents/g DW	14.39 ± 0.43
Ash Content	%	30.27 ± 0.43
Carbon Content	%	39.76 ± 0.53
Nitrogen Content	%	1.04 ± 0.03
Sulfur Content	%	3.58 ± 0.10

**Table 5:** The concentration of elements in freeze-dried supernatant from *Y. lipolytica* cultures.

Element	Unit	Mean
Phosphorus (P)	mg/kg	430 ± 26.9
Potassium (K)		428 ± 31.9
Sodium (Na)		207 ± 18.4
Magnesium (Mg)		56.7 ± 3.74
Calcium (Ca)		6.34 ± 0.39
Manganese (Mn)		5.90 ± 0.18
Iron (Fe)		2.79 ± 0.19
Zinc (Zn)		2.00 ± 0.09
Aluminum (Al)		0.157 ± 0.003
Chromium (Cr)		0.082 ± 0.002
Nickel (Ni)		0.050 ± 0.001
Arsenic (As)		0.029 ± 0.007
Boron (B)		0.025 ± 0.003
Lead (Pb)		0.010 ± 0.001
Titanium (Ti)		0.009 ± 0.002
Copper (Cu)		0.006 ± 0.000
Strontium (Sr)		0.006 ± 0.000
Zirconium (Zr)		0.004 ± 0.001
Barium (Ba)		0.003 ± 0.001
Vanadium (V)		0.003 ± 0.000
Cobalt (Co)		0.001 ± 0.000

## Data Availability

The original contributions presented in this study are included in the article. Further inquiries can be directed to the corresponding authors.

## Abbreviations

The following abbreviations are used in this manuscript:

ALA	α-Linolenic acid
ANOVA	Analysis Of Variance
ARA	Arachidonic Acid
DHA	Docosahexaenoic Acid
DNS	3,5-dinitrosalicylic acid
DM	Dry Matter
DO	Dissolved Oxygen
DW	Dry Weight
EPA	Eicosapentaenoic Acid
FA	Fatty Acid(s)
FAME	Fatty Acid Methyl Ester(s)
GC	Gas Chromatography
HDL	High-Density Lipoprotein
IAA	Indole-3-Acetic Acid
ICP-OES	Inductively Coupled Plasma–Optical Emission Spectrometry
ISO	International Organization for Standardization ISO
LA	Linoleic Acid
LDL	Low-Density Lipoprotein
MUFA	Monounsaturated Fatty Acid(s)
PUFA	Polyunsaturated Fatty Acid(s)
SCO	Single-Cell Oil
SCP	Single-Cell Protein
SD	Standard Deviation
SFA	Saturated Fatty Acid(s)
UFA	Unsaturated Fatty Acid(s)
vvm	Volumes of Gas Per Volume of Liquid Per Minute
WFO	Post-frying waste oil
YPG	Yeast Extract–Peptone-Glucose (Medium)
YPD	Yeast Extract–Peptone-Dextrose (Medium)
